# SLE Single-Step Purification and HPLC Isolation Method for Sterols and Triterpenic Dialcohols Analysis from Olive Oil

**DOI:** 10.3390/foods10092019

**Published:** 2021-08-27

**Authors:** Manuel León-Camacho, María del Carmen Pérez-Camino

**Affiliations:** Lipid Characterization and Quality Department, Instituto de la Grasa, Spanish National Research Council, 41013 Seville, Spain

**Keywords:** sterols, olive oil, triterpenic dialcohols, supported liquid extraction, high performance liquid chromatography, gas chromatography

## Abstract

The unsaponifiable fraction of oils and fats constitutes a very small fraction but it is an essential part of the healthy properties of some specific oils. It is a complex fraction formed by a large number of minor compounds and it is a source of information to characterize and authenticate the oil sample. Specially, the composition of sterols of any oil or fat is a distinctive feature of itself and, therefore, it has become a useful tool for detecting contaminants and adulterants in oils. A new supported liquid extraction (SLE) technique for the analysis and characterization of the unsaponifiable fraction of fats and oils is proposed. The SLE system includes, as a stationary phase, a combination of adsorbent materials which allow a highly purified unsaponifiable matter ready to be isolated by high performance liquid chromatography (HPLC) and quantified by gas chromatography (GC). This method ensures the removal of fatty acids, avoiding possible interferences and making the analysis of sterols and triterpenic dialcohols easier. The procedure uses a small sample size (0.2 g), reduces the volume of solvents and reagents, and reduces the handling of samples subjected to analytical control. All this is achieved without losing either precision—a relative standard deviation of each compound lower than the reference value (≤16.4%)—or recovery, being for all compounds higher than 88.00%. Therefore, this new technique represents a significant economic and time saving in business control laboratories, a larger productivity and enhancement of working safety.

## 1. Introduction

The authentication of foodstuff has been developed according to market tendencies, and analytical methods have evolved to detect adulterations.

The unsaponifiable fraction of oils or fats constitutes a very small fraction but an essential part of oils’ healthy properties. It is a complex fraction formed by a large number of minor compounds. These compounds rarely represent more than 2% of the oil composition and include many compounds of a different nature [1].

In addition, the unsaponifiable matter is the most important fraction of edible fats and oils from the point of view of the characterization and verification of their authenticity. Its different compounds are used as chemical descriptors for the authentication of these products. In particular, the composition of sterols of any oil or fat is a distinctive feature of itself and, therefore, it has become a useful tool for the detection of contaminants and adulterants of oils [2,3]. In the case of olive oils, the analytical methods to determine the content in several sterols and triterpenic dialcohols and their values are described in detail in the official regulations [4,5,6,7,8].

Conventional methods for the determination of a sterol fraction consist of several steps: saponification, liquid–liquid extraction of the unsaponifiable matter, isolation of the 4-desmethylsterols, by either thin layer chromatography (TLC) or high performance liquid chromatography (HPLC), and quantification by gas chromatography with a flame ionization detector. All these procedures take a long time and require huge amounts of solvents and excessive handling [5,6,7,9,10].

The most critical step in the unsaponifiable fraction analysis is its purification to obtain the different groups of compounds. Thus, solid-phase extraction techniques have been developed to purify the unsaponifiable fraction, using C18 [11] as well as a silica cartridge [12,13] as an absorbent. However, these cartridges do not separate the unsaponifiable matter properly.

On several occasions, gas chromatography analysis of certain fractions of compounds from a more or less complex matrix, such as the entire unsaponifiable fraction, presents some difficulties, primarily when these fractions have a high number of compounds [14]. Furthermore, the performance of chemical reactions or derivatizations in order to isolate a specific fraction may cause problems due to alterations.

In that case, the previous isolation of the fraction via liquid–liquid extraction, despite being the standardized and official process [6], is not a very suitable procedure as it is tedious and takes a long time to perform. In addition, it is necessary to use a large oil sample (between 5 and 20 g) and solvent volume for the extraction (in the order of 300 mL); if emulsions (which commonly appear) come into play, it may take extra time. Finally, it is essential to eliminate the whole solvent used in the extraction; then, a purification needs to be done. Thus, before gas chromatography analysis, isolations have to be performed using thin layer chromatography, open glass column [15], solid phase extraction (SPE) [16], or high-performance liquid chromatography (HPLC) [5,7,17]. To sum up, in order to obtain the unsaponifiable fraction of an oil or fat and purify it through a conventional procedure, it is necessary to use a large sample, a high volume of organic solvents and a long period of time. All this may cause losses and contamination.

In the particular case of using a previous isolation technique, such as HPLC, it might be off-line or on-line, with a liquid chromatograph being needed in both cases to carry out the procedure. In the former case, the selected fraction is collected, the mobile phase is removed, and the fraction is transferred to the gas chromatograph using some of the already known techniques [1]. When on-line coupling techniques are used, a more or less complex interphase is required [18,19] in order to allow the transition from the liquid state (high pressure) to the gas state (low pressure); moreover, interfaces are very different depending on whether the liquid chromatography is absorbent or distributive [20,21,22].

The isolation of target compounds from unsaponifiable fractions using the aforementioned techniques is a critical step, especially in the case of olive oils, where sterols and triterpenic dialcohols elute in the same chromatographic zones, leading to incorrect results. The previous step (isolation of the unsaponifiable matter from the saponifiable fraction) is also critical, and, with this aim, in 1973 Hadorn and Zürcher [23] published one of the first attempts at isolation using column systems with a mixture of adsorbents.

An alternative to the abovementioned isolation techniques, which is not widely known, is supported liquid extraction (SLE). Basically, it consists of a chemically inert support, highly purified, used as a stationary phase to retain the aqueous phase, formed by phyllosilicates. The water is very easily adsorbed onto the surface of the phyllosilicate particles. The main phyllosilicate used in this technique is the diatomaceous earth. Johnson et al. reported this extraction method for the first time in 1997, using a calcinated diatomaceous earth called Hydromatrix [24].

SLE techniques for isolation of the unsaponifiable fraction have been developed using cartridges of diatomaceous earth Agilent Chem Elut, 20 mL, unbuffered, followed by filtering through an anhydrous sodium sulphate and an isolation of the fractions via solid phase extraction, using cartridges of activated silica with potassium, and eluting them with solutions of different ratios of hexane/diethyl ether. Next, sterol and triterpenic dialcohol fractions were derivatized and analyzed by gas chromatography [25]. 

The aim of this work was to develop a new SLE technique for the analysis and characterization of the unsaponifiable fraction of fats and oils. A SLE method that includes a stationary phase combining different adsorbent materials is presented. In addition, different solvent mixtures from that used in the literature (diethyl ether) are assayed. Finally, the use of the HPLC technique notably enhances the purification of the unsaponifiable matter, avoiding—as opposed to the described methods in the literature—interference from other unsaponifiable compounds. It will reduce sample size, the volume of solvents and reagents, and the handling of samples subjected to analytical control. Without losing precision and recovery while saving time and resources, this new technique will turn provide higher productivity and enhance working safety for business control laboratories.

## 2. Materials and Methods

### 2.1. Reagents and Solutions

Ethyl acetate and n-hexane of LiChrosolv grade were supplied by Merck (Darmstadt, Germany). Ethanol (96% vol.) and diethyl ether of analytical grade were supplied by VWR (Leuven, Belgium). Potassium hydroxide (85%) pellets and anhydrous sodium sulphate, both of PA-ACS grade, were supplied by Panreac (Barcelona, Spain). 2,7-diclorofluorescein of analytical grade was supplied by Fluka Chemical Co. (Ronkonkoma, NY, USA). 5α-cholestane-3β-ol and betulin were supplied by Fluka Chemical Co. (Ronkonkoma, NY, USA) and used as internal standards. Derivatizing reagent, a mixture 99:1 (*v*/*v*) of N,O-bis (trimethylsilyl)-trifluoroacetamide and trimethylchlorosilane were supplied by Tokyo Chemical Industry CO. (Tokyo, Japan). Anhydrous pyridine of analytical grade (ref. 7463) was supplied by Merck (Darmstadt, Germany). Diatomaceous Earth, 6/60 mesh was supplied by Restek (Bellefonte, PA, USA). Adsorbent-phase Bondesil-NH2 40 µm was supplied by Varian, Inc., (Walnut Creek, CA, USA). Commercial SLE cartridge Strata DE, 60 cc, was supplied by Phenomenex (Torrance, CA, USA). All other reagents were of analytical grade.

### 2.2. Samples

Virgin olive oil from the cultivar variety Picual and refined olive pomace oil were used. The virgin olive oil sample was obtained from the oil mill pilot plant located in the “Instituto de la Grasa (CSIC)”, operating in the usual conditions, during the season 2018/2019. Refined olive pomace oil and refined sunflower seed oil samples were supplied by a local refining industry. The olive oil samples were homogenized and divided into aliquots to carry out the different assays. Additionally, a refined sunflower oil sample was purchased from a local store. The unsaponifiable matter was extracted using the different methods described in the sections below.

### 2.3. Instrumentation

#### 2.3.1. HPLC Isolation

Sterols and dialcoholic triterpenic fractions were isolated by HPLC. The HPLC system consisted of an Agilent (Palo Alto, CA, USA) 1200 series liquid chromatograph, equipped with a micro vacuum degasser, a binary pump, an auto-sampler injector provided with a preparative-head assembly of 900 µL, a Peltier furnace, a refractive index detector 1100 series and an analytical fraction collector installed at the exit of the detector for the recovery of the sterol fraction. A chemical station HP was used for controlling and monitoring the system. The separation was performed in a 150 mm × 3.9 mm, particle size 4 µm Nova Pak Silica 60 Ȧ Water Millipore Corporation (Milford, MA, USA) column. The temperature of the column and the detector were held, respectively, at 20 and 35 °C. The mobile phase was n-hexane/ethyl acetate 90/10 (*v*/*v*). The flow rate was established at 0.6 mL·min^−1^ for 30.00 min.

#### 2.3.2. Gas Chromatography-Flame Ionization Detector (GC-FID) Analysis

The collected fraction using the HPLC system was analyzed in an Agilent (Palo Alto, CA, USA) 7890A gas chromatograph equipped with a split/splitless injector and a flame ionization detector; a capillary HP-5MS column (30 m × 0.25 mm I.D., 0.25 µm film thickness, Agilent J & W, Palo Alto, CA, USA) and an Agilent G 4513A automatic injector were used. The oven temperature was kept at 265 °C isothermally. The operating condition of injector was split mode and its temperature was kept at 310 °C, while the detector temperature was 310 °C and the injection volume was 1 µL. Hydrogen was used as the carrier gas at 1.0 mL·min^−1^ in constant flow mode and a split ratio of 1:10. Air and hydrogen at flow rates of 300 and 30 mL·min^−1^, respectively, were used for the detector, which had an auxiliary flow of 30 mL·min^−1^ of nitrogen.

#### 2.3.3. ATR-FTIR Spectroscopy

A Bruker 55 Equinox S FTIR spectrometer with a DGTS detector (Bruker Optics, Ettlingen, Germany) was used in this study. The sampling station was equipped with an overhead, detachable attenuated total reflectance (ATR, six bounces, Specac, Orpington, UK) accessory consisting of a zinc selenide crystal mounted in a shallow channel for the sample containment. Each spectrum was recorded at room temperature in the region of 4000–600 cm^−1^ by an average of 50 scans at a resolution of 4 cm^−1^.

### 2.4. Sample Treatment

#### 2.4.1. IOC and EU Methods (Liquid–Liquid Extraction Method)

The preparation and analysis of the unsaponifiable matter were carried out in accordance with the IOC and EU methods of analysis (official methods) [5,6,7]. The oil samples, with added 5α-cholestane-3β-ol as an internal standard, were saponified with potassium hydroxide 2 M in ethanolic solution (with 20% of water), and the unsaponifiable fractions were then extracted three times with diethyl ether.

The 4-desmethylsterol and triterpenic dialcohol fractions were extracted, as has been previously described in the literature [5,6,7]. Briefly, 5.00 ± 0.10 g of the oil sample was weighed in a flask containing 5α-cholestane-3β-ol (0.5 mL of a solution of 0.01% *m*/*v* in ethyl acetate was previously added and evaporated until dryness). Then, the oil sample containing the internal standard was saponified for 60 min with 50 mL of 2 M ethanolic potassium hydroxide with 20% water. The solution was passed into a 500 mL decanting funnel, 100 mL of distilled water was added and the mixture was extracted twice with three 80 mL portions of diethyl ether. The organic extracts were combined in another funnel and washed several times with 100 mL portions of water until the wash reached neutral pH. The diethyl ether solution was dried over anhydrous sodium sulphate and evaporated to dryness in a rotary evaporator at 30 °C under reduced pressure.

The complete unsaponifiable dried fraction was then redissolved in approximately 3.00 mL of the mobile phase, and 250 µL of the solution was injected into the HPLC system as described in the Instrumentation section. Subsequently, the fraction that eluted from minutes 11.00 to 24.00 was recovered through the analytical fraction collector. The solvent was evaporated to dryness under reduced pressure. The 4-desmethylsterol and triterpenic dialcohol fractions were treated with 150 µL of the derivatizing reagent to obtain the trimethyl silyl derivates for subsequent GC-FID analysis.

#### 2.4.2. Proposed Method (Supported Liquid Extraction Method)

The internal standard solution (40 µL of α-cholestanol for virgin olive oil and 100 µL for refined olive pomace oil, and 100 µL of Betulin for both samples) was introduced into a 4 mL vial and the solvent was evaporated under a N2 stream. Next, 0.300 ± 0.010 g of the virgin oil sample or 0.200 ± 0.010 g of the olive pomace oil were weighed in the same vial which contained the standard. One milliliter of an ethanolic solution of KOH 2 M was added to the vial, and it was closed and heated up in a thermo-block for 45 min at 85 °C, shaking the vial every 15 min.

Once the time was over, 2 mL of distillate water was added to the vial and the content was poured into a homemade prepared column of 20 mm I.D. filled with 1 g of amine (lower layer) and 5 g of diatomaceous earth (upper layer) of particle size <0.5 mm, obtained by sifting as described in the Reagents and Solutions section, leaving the mixture for at least ten minutes before eluting. Then, a volume of 45 mL of a hexane:ethyl acetate mixture (85:15, *v*/*v*) was passed through the cartridge. The whole content was collected in a 50 mL flask and evaporated to dryness in a rotary evaporator at 30 °C under reduced pressure; then, it was dissolved again with 300 µL of the mobile phase. Next, the content was centrifuged at 14,000× *g* for approximately 1 min. The upper layer was collected with a pipette, poured into a HPLC vial and 250 µL was injected into the chromatograph. Subsequently, as described in the IOC or EU method, the fraction that eluted from minutes 11.00 to 24.00 was collected, evaporated, silylated with 150 µL of derivatizing reagent, and injected into the GC-FID.

**Figure 1:**
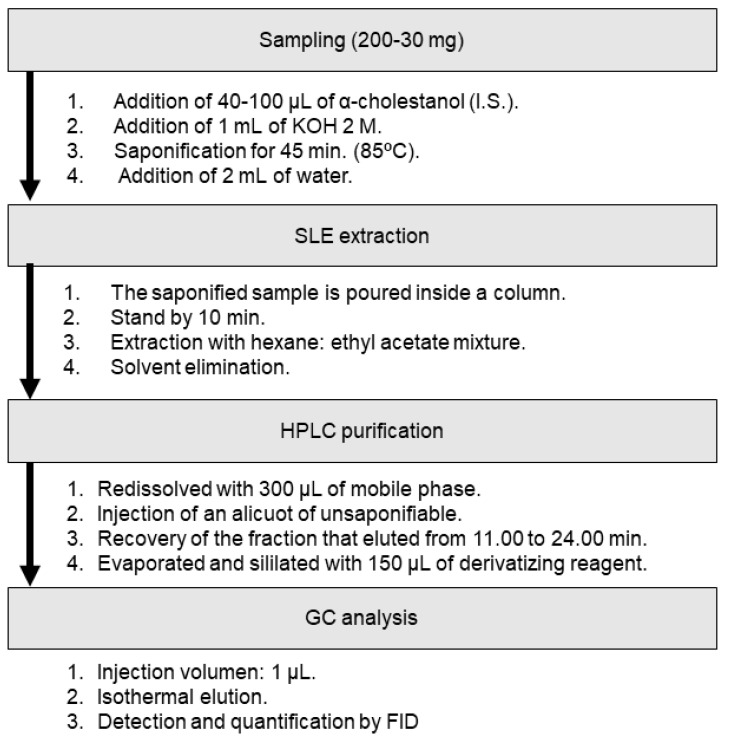
Workflow for the determination of sterols and triterpenic dialcohols in olive or olive pomace oils.

Figure 1 details the whole procedure for the determination of sterol and triterpenic dialcohol fractions using the proposed method: first of all, the saponification was performed, then the sample extraction by SLE, HPLC isolation, derivatization and, finally, GC-analysis.

### 2.5. GC-Data Analysis

The GC-peak areas were calculated with Agilent ChemStation OpenLAB, and the determination of individual 4-desmethylsterols and triterpenic dialcohols was carried out by evaluating the corresponding relative percentage according to the normalization area procedure assuming an equal factor response for any species. The quantitative determination of the total sterols and triterpenic dialcohols were performed relative to the peak area of the known concentration of the internal standard.

### 2.6. ATR-FTIR Spectra

IR spectra were acquired for the unsaponifiable matter obtained by the official method as well as by the suggested method, according to the described method by Tena et al. [26].

### 2.7. Recovery and Precision

In order to study the recovery and reproducibility of the present method, a complementary experiment was carried out. Recovery data were calculated, comparing the results obtained from the proposed and the official methods. Six replicates were made in each case. For the determination of reproducibility, the replicates were performed on different days and in the same laboratory [27].

## 3. Results and Discussion

Among all the possibilities for the determination of sterols in vegetable oils, gas chromatography with a FID detector is the last step in the process of their quantification and, it is, with minor differences, common to all proposals. However, the previous steps are key to performing their determination accurately, and there are important differences between the different proposed methods as well as with respect to the official methods.

### 3.1. Saponification, SLE and HPLC Isolation

The first step for the sterols’ determination, the saponification, is mandatory in all of the methodologies described, as these unsaponifiable compounds are present in a free, and esterified with fatty acids, form. Therefore, for their whole determination, they must be transformed and isolated in the form of free sterols. 

In the regulations, after one hour of boiling, the unsaponifiable matter can be isolated from the saponified one. For that purpose, the official methods [5,6,7] described several liquid–liquid extractions with diethyl ether where the unsaponifiable fraction is extracted almost free of the saponified part, which is solubilized into the water. Next, a cleaning step of the collected diethyl ether fractions with distilled water is necessary to guarantee the absence of the basic reagent used for the saponification process and the almost complete absence of the saponified matter. As can be deduced, large volumes of solvent and time are consumed. The proposed method here described starts with the saponification of an oil sample about sixteen times smaller than that used in the official method; it is saponified for 45 min and is passed through a SLE column.

Table 1 shows the details of the sample quantities, water addition, volumes, washes, drying of the sample and purification that are carried out in the proposed procedure compared to the regulation and to another process recommended by a commercial company [17]. Among others, the main differences are the sample amounts and the solvent used for the extractions. As can be seen, when using SLE, the volume of the sample is reduced by 12–25 times compared to L–L, and the volume of the solvent is also reduced by more than six times. All this makes the official method long and tedious, where, in addition, the formation of emulsions that must be broken is frequent.

For all of this, the SLE is, at present, a rapid and the best alternative to the L–L extraction used in the official method for isolating the unsaponifiable matter. Thus, Table 2 presents a detailed comparison of the operation times used in each of the steps in the L–L (official methods) and SLE methods; also, the proposed method is compared to that proposed by the commercial company [17]. As can be observed, and considering all the steps, the new procedure is more than twice as fast as the official one, which is an important advance in work per day, and 1.66 times faster than the current commercial method [17]. Furthermore, as Table 1 shows, the number of steps in the suggested method is reduced to 57%, regarding the official method, which notably reduces losses and pollution during the process.

The SLE used here combines diatomaceous earth with particle size <0.5 mm with a layer of amino phase in the same cartridge. This blend guarantees an unsaponifiable matter sample free of water and free fatty acids, which is of great importance for the good isolation of each unsaponifiable component and particularly the sterols and triterpenic dialcohols in the following step. On the other hand, the use of diethyl ether, acting as an extraction solvent as proposed in the regulation and commercial methods, extracts the unsaponifiable matter together with some soaps, which interfere in the purification of the unsaponifiable matter and must be eliminated with water addition. Nevertheless, this inconvenience is avoided by using the admixture hexane:ethyl acetate here proposed.

Once the unsaponifiable matter has been obtained, it is necessary to isolate, by means of a chromatographic technique (TLC or HPLC), the series of compounds of interest—in our case, the sterols and triterpenic dialcohols. This step is almost mandatory to obtain better precision in the quantification, as other unsaponifiable compounds, such as alcohols, tocopherols or hydrocarbons, interfere.

In the official regulations, TLC has been used for years as the best method for the isolation, but at present, an HPLC method is also proposed for it. Before its recent inclusion in the regulations of the European Union and IOC in 2020 [5,7], the HPLC method for sterol isolation was studied by various different authors [1,16,28].

With the selected conditions studied here, in the HPLC chromatogram of the unsaponifiable matter corresponding to the oil samples, the fraction collected ranged from Δ^5^- and Δ^7^-sterols to erithrodiol+Uvaol.

The RP-HPLC method proposed for the isolation is more precise and less time-consuming, and many other factors indicate that the HPLC procedure is better than TLC. Thus [29], reported that the insufficient separation in TLC between the band of sterols and triterpenic alcohols, the delimitation of the band of sterols in the TLC and the impurities close to the band of triterpenic dialcohols are the main causes for the lack of precision in the determination of Δ^7^-sterols.

On the other hand, the eluent admixture hexane-diethyl ether (65:35 *v*/*v*) at the flow rate proposed by official methods (EU, IOC) [16] was replaced here by n-hexane/ethyl acetate 90/10 (*v*/*v*) at a flow rate of 0.6 mL·min^−1^; the use of diethyl ether presents high pressure and burble problems in the HPLC equipment.

The amount and size of diatomaceous earth particles used to prepare the columns is extremely important, due to the fact that these parameters determine the amount of absorbed water and water flux through the column; in the specific case of the proposed method, 5 g of diatomaceous earth with a particle size <0.05 mm was used. Table 3 shows the differences between the SLE columns in the suggested and in the commercial method [17]; the amount of diatomaceous earth used in the suggested method is only 5 g, compared to 19 g of this material in the commercial columns, which is three times greater in volume.

**Figure 2:**
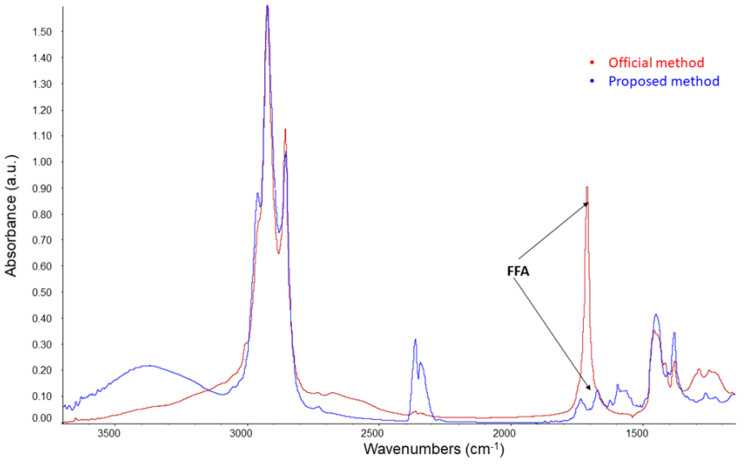
Spectra ATR—FTIR of the unsaponifiable matter from virgin olive oil obtained by official method and proposed method.

This newly suggested method does not require the drying and free fatty acid removal steps, in contrast to methods described in the literature [17,25], because using ethyl acetate and hexane in conjunction with the type of column minimizes the amount of water and soap extracted to trace levels. Regarding the removal of possible free fatty acids from hydrolysis of extracted soap traces, it takes place in the same SLE column as that in which 1 g of adsorbent phase Bondesil-NH_2_ 40 μm is deposited. In order to check what is mentioned above, ATR-FTIR spectra were examined for unsaponifiable compounds of a virgin olive oil obtained via the official method as well as via the suggested method, in both cases before the purification step by HPLC. As Figure 2 shows, for the unsaponifiable matter obtained through the official method, there is a high-intensity band around a wavelength of 1716 cm^−1^ in the infrared spectra, which corresponds to the free fatty acids [26,30], whereas this band is almost negligible for the unsaponifiable matter obtained through the suggested method.

The HPLC technique was used to purify the unsaponifiable fraction. A chromatographic or solid phase extraction technique [5,16,25] is needed in the cases of olive oil and olive pomace oil in order to remove interferences that might appear when analyzing, through the GC technique, the sterol fraction with specific triterpenic alcohols and methyl sterols, among other reasons. However, the official methods do not use any purification techniques before HPLC isolation.

The official methods use mixtures of hexane/diethyl ether 50:50 (*v*/*v*) as the mobile phase; using diethyl ether in HPLC may cause bubble and pressure oscillation issues. However, these issues may be avoided by using mixtures of hexane/ethyl acetate 90:10 (*v*/*v*) at lesser fluxes, as in the suggested method. Moreover, Si columns with a particle size of 5 µm and dimensions of 250 mm × 4.6 mm might be substituted by columns with dimensions of 150 mm × 3.9 mm and a particle size of 4 µm; thanks to this modification, resolution is improved, obtaining a lesser peak width.

**Figure 3:**
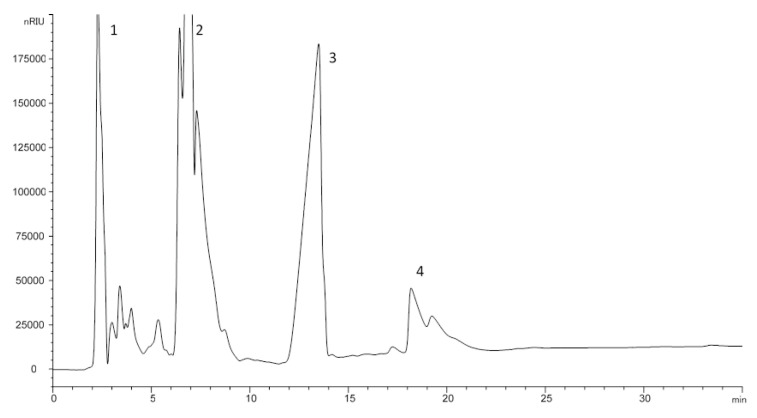
HPLC chromatogram of the unsaponifiable fraction from olive pomace oil. 1: Aliphatic and terpenic hydrocarbons; 2: linear and triterpenic alcohols and methyl sterols; 3: sterols; 4: triterpenic dialcohols.

Figure 3 shows a HPLC chromatogram of the unsaponifiable fraction from the refined olive pomace oil sample according to the suggested method. As can be observed, four groups of compounds are clearly differentiated: aliphatic and terpenic hydrocarbons (1), linear and triterpenic alcohols and methyl sterols (2), sterols (3) and triterpenic dialcohols (4). Each one can be recovered in an established time interval; in this work, only the sterol and triterpenic dialcohol fractions were studied, which may be recovered between 11 and 24 min without interferences from methyl sterols and triterpenic alcohols. This interval is relatively similar to that suggested in the official methods; however, as can be observed in the figure, the resolution between the different groups of compounds is better in the case of the suggested method, because the unsaponifiable matter is not purified in the official methods to guarantee the absence of free fatty acids.

**Figure 4:**
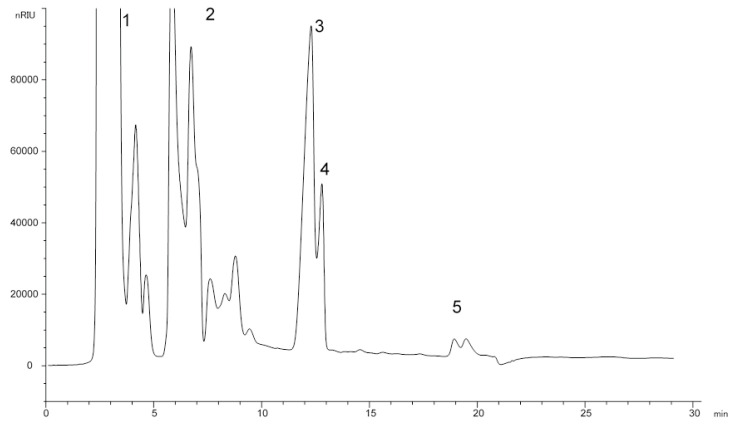
HPLC chromatogram of unsaponifiable fraction from a mixture of olive pomace oil and sunflower seed oil. 1: Aliphatic and terpenic hydrocarbons; 2: linear and triterpenic alcohols and methyl sterols; 3: Δ^5^-sterols; 4: Δ^7^-sterols; 5: triterpenic dialcohols.

Figure 4 shows a HPLC chromatogram of the unsaponifiable fraction from refined sunflower seed oil. As this figure shows, within the chromatographic conditions proposed in the present work, a good resolution between sterols with Δ^5^- and Δ^7^-sterols structure can be obtained.

### 3.2. Gas Chromatography Determination of Sterols and Dialcoholic Triterpenes

**Figure 5:**
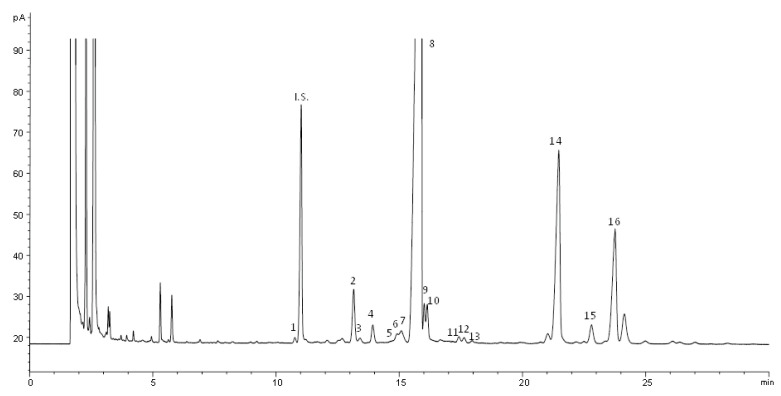
GC—FID chromatogram of olive pomace oil sterols fraction recollected from HPLC chromatograph. 1: cholesterol; 2: campesterol; 3: campestanol; 4: stigmasterol; 5: Δ^7^-campesterol; 6: Δ^5,23^-stigmastadienol; 7: clerosterol; 8: β-sitosterol; 9: sitostanol; 10: Δ^5^-avenasterol; 11: Δ^5,24^-stigmastadienol; 12: Δ^7^-stigmastenol; 13: Δ^7^-avenastol; 14: erythrodiol; 15: uvaol; 16: betulin.

Figure 5 shows a gas-chromatogram corresponding to sterols and triterpenic dialcohols obtained from a refined olive pomace oil. Sixteen peaks are numbered and correspond to the compounds, which eluted in the same order as specified in Table 4. The chromatographic profile matches with the chromatograms published in the regulations for virgin and refined olive oils, and as the conditions are the specified and recommended in the regulation, β-sistosterol elutes in the range of 20 ± 5 min. The internal standard allows the quantitative determination of all the sterols and triterpenic dialcohols, and is well separated from all the peaks, including the nearest, the cholesterol eluting just ahead of it. It is noteworthy that it is a refined olive oil, since just before the clerosterol peak, the Δ^5,23^-stigmastadienol elutes, only present when the oil has been refined. In addition, the TD, erythrodiol and uvaol are undoubtedly identified by their magnitude, as corresponds to an olive pomace oil, but also because their relative retention times with respect to β-sistosterol are 1.41 and 1.52 for erythrodiol and uvaol, respectively, as specified in the regulation.

If any of the above-mentioned purification techniques are not employed, it is hard to avoid interferences. Some authors have suggested a longer analysis time in GC in order to avoid such interferences [17]. However, this procedure has low efficiency because it increases the retention of the compounds inside the column, increasing diffusion and, therefore, reducing sensibility and precision.

### 3.3. Validation of the Method

The proposed procedure was validated in house by performing six replicated analyses of two different olive oil samples. The mean and the standard deviation were calculated, and the data are reported in Table 4. Furthermore, accuracy was evaluated, and the results are also included. It can be seen that the repeatability is good in all cases because the values of the relative standard deviations are lower than the reference value derived from the Horwitz equation [31] (RSDH = 16.4%). Therefore, the results for different sterols and triterpenic dialcohols indicate a good repeatability for the assay.

In order to evaluate the recovery of the proposed method, the mean values of the different compounds obtained from six replicates were compared to the results obtained by the procedure specified in the official methods (Table 5). As can be observed in the mentioned table, all compounds showed recovery values higher than 88.00%, which means a good recovery was obtained in all cases.

## 4. Conclusions

The analytical method proposed for isolating and quantifying the unsaponifiable fraction is based on SLE extraction, and it may be proposed as a good alternative to the liquid–liquid extraction methods (official methods), since it offers a clear advantage over the recovery of the sterol and triterpenic dialcohol fractions, and the results obtained are not significantly different from those obtained by the official methods.

The new SLE/HPLC method detailed in this work allows a rapid and highly accurate separation of the different compound families that are part of the unsaponifiable matter from olive oils. The isolation of the unsaponifiable fraction proposed makes the analysis of the compounds included in this fraction (sterols, aliphatic alcohols, tocopherols, etc.) easier and less time-consuming than those previously reported. Thus, the analysis time was reduced by more than half, and the volume of solvent used was also reduced by more than six times with respect to the official methods. 

It has been demonstrated that the methodology based on an off-line combination of HPLC and GC-FID is a good, quick and reproducible analytical method for the isolation and quantification of the sterols and triterpenic alcohols in olive oils. The precision and accuracy of the procedure described have been checked, showing a high recovery of the different compounds studied.

Furthermore, this method ensures the removal of fatty acids, avoiding all the possible interferences during the GC quantification.

The results obtained enable the assessment of the olive oil’s quality in accordance with UE and IOC sterol criteria and agree with the mentioned regulations.

## Figures and Tables

**Table 1:** Details of the procedures carried out for the isolation of the sterol and triterpenic dialcohol fractions.

Steeps	L–L Method	SLE Methods	
	Official Methods	Commercial [17]	Proposed Method
Sample amount	5 g	0.2–0.4 g	0.3 g
Water addition	100 mL	13.5 mL	2 mL
Extraction	3 × 100 mL	3 × 15 mL	45 mL
Washed	3 × 50 mL	no	no
Dryed	30–50 g Na_2_SO_4_	SPE Na_2_SO_4_	no
FFAs removal	KOH _in TLC Si_	KOH _in SPE Si_	no
Purification	TLC or HPLC	no	HPLC

**Table 2:** Comparison of the operation times (min.) for the L–L and SLE methods.

Steeps	L–L Method	SLE Methods	
	Official Methods	Commercial [17]	Proposed Method
Sampling and Internal standard addition	7:00	1:20	1:20
KOH 2M training and addition	3:11	1:00	1:00
Saponification	60:00	50:00	45:00
Water addition and cooling	30:00	15:00	10:00
Extraction with organic solvent	15:00	5:00	5:00
Washed	20:36	no	no
Dryed	20:36	15:00 SPE Na_2_SO_4_	no
Free Fatty Acids removal	17:25	15:00 SPE Si, KOH	no
Purified + derivatization	50:00	30:00	30:00
GC Analysis	30:00	70:00	30:00
TOTAL time	253:48	202:20	122:20

**Table 3:** Comparison between SLE columns.

	Commercial [17]	Proposed Method
Volume (mL)	75	25
Stuffing amount (g)	19.0	5.0
Length (cm)	14.0	8.5
Sorbent	Unknown	diatomaceous earth

**Table 4:** Reproducibility of the sterols and triterpenic dialcohols of oil samples by the proposed SLE method and official methods.

	VOO						OPO					
	SLE			OM *			SLE			OM *		
	Means ** N = 6	SD	RSD (%)	Means ** N = 6	SD	RSD (%)	Means ** N = 6	SD	RSD (%)	Means ** N = 6	SD	RSD (%)
Cholesterol	nd	nd	nd	nd	nd	nd	0.20	0.03	15.00	0.15	0.03	20.36
Campesterol	2.83	0.07	2.47	2.81	0.04	1.42	2.63	0.03	1.07	2.56	0.01	0.50
Stigmasterol	0.74	0.07	9.46	0.68	0.05	7.35	0.89	0.06	7.01	0.95	0.02	1.61
Δ^5,23^-stigmastadienol	nd	nd	nd	nd	nd	nd	0.55	0.02	3.93	0.51	0.00	0.88
Clerosterol	1.04	0.05	4.81	1.06	0.1	9.43	0.86	0.02	1.89	0.86	0.02	2.42
β-sitosterol	83.95	1.07	1.27	83.96	1.75	2.08	74.75	0.20	0.27	73.85	0.45	0.60
Sitostanol	0.69	0.03	4.35	0.78	0.04	5.13	1.45	0.11	7.38	1.44	0.06	4.23
Δ^5^-avenasterol	7.37	0.18	2.44	7.34	0.18	2.45	1.48	0.03	2.27	1.49	0.12	7.87
Δ^5,24^-stigmastadienol	0.70	0.04	5.71	0.61	0.07	11.48	1.31	0.04	2.81	1.46	0.02	1.71
Δ^7^-stigmastenol	0.42	0.04	9.52	0.38	0.04	10.53	0.35	0.03	8.57	0.31	0.04	12.90
Δ^7^-avenasterol	0.74	0.07	9.46	0.66	0.07	10.61	0.28	0.01	3.57	0.35	0.01	2.13
Erythrodiol + Uvaol ***	1.52	0.06	3.95	1.72	0.04	2.33	15.25	0.29	1.88	16.07	0.56	3.51
Total sterols (ppm)	1231.38	30.21	2.45	1224.19	30.73	2.51	5529.87	309.19	5.59	5183.04	57.45	1.11

VOO: Virgin olive oil; OPO: olive pomace oil; * official methods; nd: non detected; ** the mean data are presented as percentages of total sterols, without include the triterpenic dialcohols; *** the mean data are presented as percentages of total sterols; SD: Standard deviation; RSD: Relative standard deviation.

**Table 5:** Recovery percentages of the sterols and triterpenic dialcohols of VOO and OPO, in relation to the official method (100%). (*N* = 6).

	VOO	OPO
Cholesterol	nd	133.33
Campesterol	100.71	102.73
Stigmasterol	108.82	93.68
Δ^5,23^-stigmastadienol	nd	107.84
Clerosterol	98.11	100.00
β-sitosterol	99.99	101.22
Sitostanol	88.46	100.69
Δ^5^-avenasterol	100.41	99.33
Δ^5,24^-stigmastadienol	114.75	89.73
Δ^7^-stigmastenol	110.53	112.90
Δ^7^-avenasterol	112.12	80.00
Erytrodiol + Uvaol	88.37	94.90
Total sterols (ppm)	100.59	106.69

nd: non detected.

## Data Availability

Not applicable.
